# *Insulin2*^Q104del^ (Kuma) mutant mice develop diabetes with dominant inheritance

**DOI:** 10.1038/s41598-020-68987-z

**Published:** 2020-07-22

**Authors:** Daisuke Sakano, Airi Inoue, Takayuki Enomoto, Mai Imasaka, Seiji Okada, Mutsumi Yokota, Masato Koike, Kimi Araki, Shoen Kume

**Affiliations:** 1 School of Life Science and Technology, Tokyo Institute of Technology, 4259-B-25 Nagatsuta-cho, Midori-ku, Yokohama, Kanagawa 226-8501 Japan; 2 Laboratory of Developmental Genetics, Institute of Resource Development and Analysis, Chuo-Ku, Honjo 2-2-1, Kumamoto, 860-0811 Japan; 3 Division of Hematopoiesis, Joint Research Center for Retroviral Infection, Kumamoto University, Honjo 2-2-1, Kumamoto, 860-0811 Japan; 4 Department of Cell Biology and Neuroscience, Juntendo University Graduate School of Medicine, Hongo 2-1-1, Bunkyo-ku, Tokyo, 113-8421 Japan; 5 Present Address: Department of Genetics, Hyogo College of Medicine, 1-1 Mukogawa-cho, Nishinomiya, Hyogo 663-8501 Japan

**Keywords:** Biotechnology, Genetics

## Abstract

Insulin gene mutations have been identified to cause monogenic diabetes, and most of which developed permanent neonatal diabetes at young ages before 6 months of age in humans. To establish an animal model of permanent diabetes, we performed genome editing using the CRISPR/Cas9 system. We generated a novel Kuma mutant mice with p.Q104del in the *Insulin2* (*Ins2*) gene in a BRJ background that exhibits a severe immune deficiency. Kuma mutant mice are non-obese and developed hyperglycemia from 3 weeks after birth in both males and females, which are inherited in a dominant mode. Kuma mutant mice displayed reduced insulin protein levels from 3-weeks-old, which seem to be caused by the low stability of the mutant insulin protein. Kuma mutant showed a reduction in islet size and islet mass. Electron microscopic analysis revealed a marked decrease in the number and size of insulin granules in the beta-cells of the mutant mice. Hyperglycemia of the mutant can be rescued by insulin administration. Our results present a novel insulin mutation that causes permanent early-onset diabetes, which provides a model useful for islet transplantation studies.

## Introduction

Insulin is secreted from the endocrine pancreas that functions to regulate blood glucose homeostasis. Neonatal diabetes is a monogenic form of diabetes resulting from mutations in a number of genes, which encode protein products that play a role in the function of the pancreatic beta-cells. Among these genes, Insulin gene mutations have been identified to cause permanent neonatal diabetes in humans^1–4^.

In the mice, Akita *Insulin2* (*Ins2*)^C96Y^ and Munich *Ins2*^+/C95S^ mouse model bearing insulin gene mutations are reported to develop early-onset diabetes that displays an autosomal dominant mode of inheritance^5, 6^. These mice models show hyperglycemia that does not accompany with obese or insulitis and exhibit pancreatic beta-cell dysfunction as a primary defect. The phenotypes resemble permanent neonatal diabetes in the human and therefore serve as a widely used model for the studies of beta-cell dysfunction and a model for insulin supplementation and transplantation studies. Experimental animals such as pig model of permanent neonatal diabetes have been generated by introducing an *INS*^C94Y^ transgene into the fertilized egg^7^.

We aimed to establish a mouse model exhibiting severe insulin-deficiency and beta-cell dysfunction under a severe immunodeficiency background so that the mouse model could be used for efficacy studies of human stem cell-derived or xenogeneic beta-cell transplantations without considerating the immune responses. We first attempted to introduce mutation in *Ins2* gene into severe immune-deficient BRJ (*Rag-2*/*Jak3* double-deficient in BALB/c background) mice lacking mature T and B lymphocytes and NK cells^8^. As a result, we generated an immune-deficient mice model of permanent diabetes bearing a mutated *Ins2* gene (Ins2 p.Q104del). The *Ins2*^+/Q104del^ mouse is named as Kuma mouse. The Kuma mouse is useful as a model for studies of beta-cell failure through beta-cell cytotoxicity and also as a model for evaluation of interspecies transplantation studies.

## Results

### Generation of diabetic Kuma mice under BRJ background

**Figure 1:**
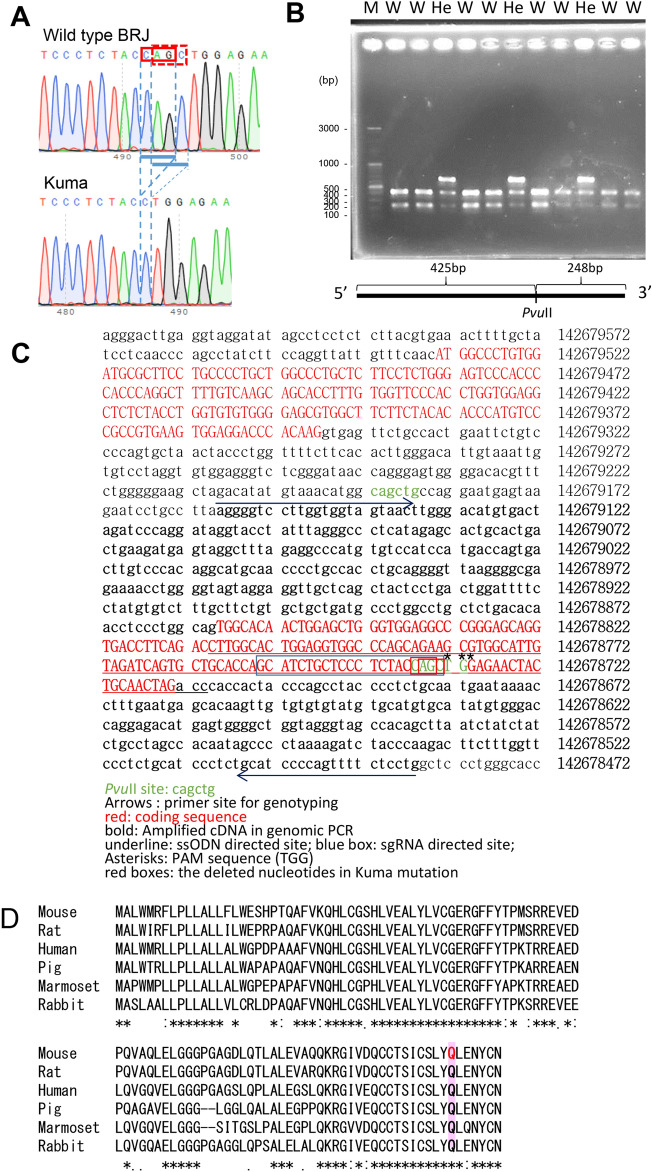
Establishment of diabetic *Ins2*^+/Q104del^ (Kuma) mice. Kuma mutation is generated under a BRJ background. Kuma mouse reveals the deletion of 3 base pairs (CAG or AGC; c.310_312del or c.311-313). Genotyping of Kuma mutant can be performed by genomic PCR and Pvu II restriction enzyme digestion. (**A**) Sequencing of wild type and Kuma mice revealed a 3 bp CAG or AGC deletion of Kuma mice. (**B**) Kuma mutation could be identified by genomic PCR, followed by restriction enzyme Pvu II digestion. Wild type fragment is digested by Pvu II and yields 425 bp and 248 bp bands. While in the Kuma mutant mice, the Pvu II site is lost, so that Kuma heterozygous mutant showed an additional larger band at 670 bp. (**C**) The sequence of the *Ins2* gene in the Kuma mutant is shown. The primer pair used is indicated by forward and reverse arrows under the primer sites shown with underlines. Red capital letters indicate the coding sequence (CDS). The sequence of the fragment amplified by genomic PCR is shown in bold letters. Two Pvu II recognition sites are marked by green letters. The Kuma mutation (AGC deletion) site is shown by a red box. (**D**) Multiple sequence alignment of mouse insulin-2 preproprotein (accession number: NP_032413.1), rat insulin-2 preproprotein (accession number: NP_062003.1), human insulin preproprotein (accession number: NP_000198.1), pig insulin isoform X1 (accession number: XP_020936937.1), marmoset insulin preproprotein (accession number: XP_002755713.1) and rabbit insulin proteins (accession number: NP_001075804.1) using Clustal W method. An asterisk (*) indicates positions which have a single, fully conserved residue. A colon (:) indicates conservation between groups of strongly similar properties—scoring > 0.5 in the Gonnet PAM 250 matrix. A period (.) indicates conservation between groups of weakly similar properties—scoring ≤ 0.5 in the Gonnet PAM 250 matrix.

To enable transplantation of human cells into mice, we attempted to introduce a mutation in the *Ins2* gene into a BRJ mouse, which exhibits severe immune deficiency^8^. We utilized gene editing using the CRISPR/Cas9 system^9, 10^ and originally designed to introduce a mutation to yield *Ins2*^+/C96Y^ (Akita) mutation. We introduced by electroporation of a 100-base (b) single-stranded oligodeoxynucleotides (ssODNs)^11^, in vitro transcribed Cas9 mRNA and a gRNA into fertilized BRJ mouse eggs to target *Ins2* gene with an Akita mutation^12^. However, instead of obtaining Akita mutation, we obtained a 3 base pair (bp) (CAG or AGC) deletion in *Ins2* gene (c.310_312del or c.311-313) that both resulted in a Gln (Q) deletion (p.Q104del) (Fig. 1A). F1 female mice revealed polyuria, which is one of the symptoms of diabetes. We named the *Ins2*^+*/*Q104del^ mutation as Kuma mutation. For genotyping of the mutant mice, we established a genomic polymerase chain reaction (PCR) system to amplify a fragment containing a Pvu II restriction enzyme site that is lost in the p.Q104del mutant (Fig. 1B). Since the mutant allele is resistant to Pvu II digestion, a larger band of 670 bp could, therefore, be detected in the mutant allele (Fig. 1B). The genomic sequence flanking the mutation site is shown in Fig. 1C. The deleted 3 bp, either CAG or AGC, are highlighted in red boxes. The sequence targeted by the ssODN or sgRNA is shown by underline or in a blue box, respectively (Fig. 1C). The deleted 3 nucleotides (nt) of the Kuma mutation are located at the 3′ of the sgRNA, immediately next to Protospacer adjacent motif (PAM) sequence (Fig. 1C). This 3nt deletion is thought to be generated by non-homologous end joining (NHEJ) caused by a double-strand break (DSB) instead of homologous recombination with the introduced ssODN. We sequenced and found that the p.Q104del is the only mutation in *Ins2* cDNA in Kuma mice. Comparison of the amino acid sequences of insulin across species, such as mouse, rat, human, pig, marmoset, and rabbit, revealed that this Q104 is a highly conserved residue (Fig. 1D).

**Figure 2:**
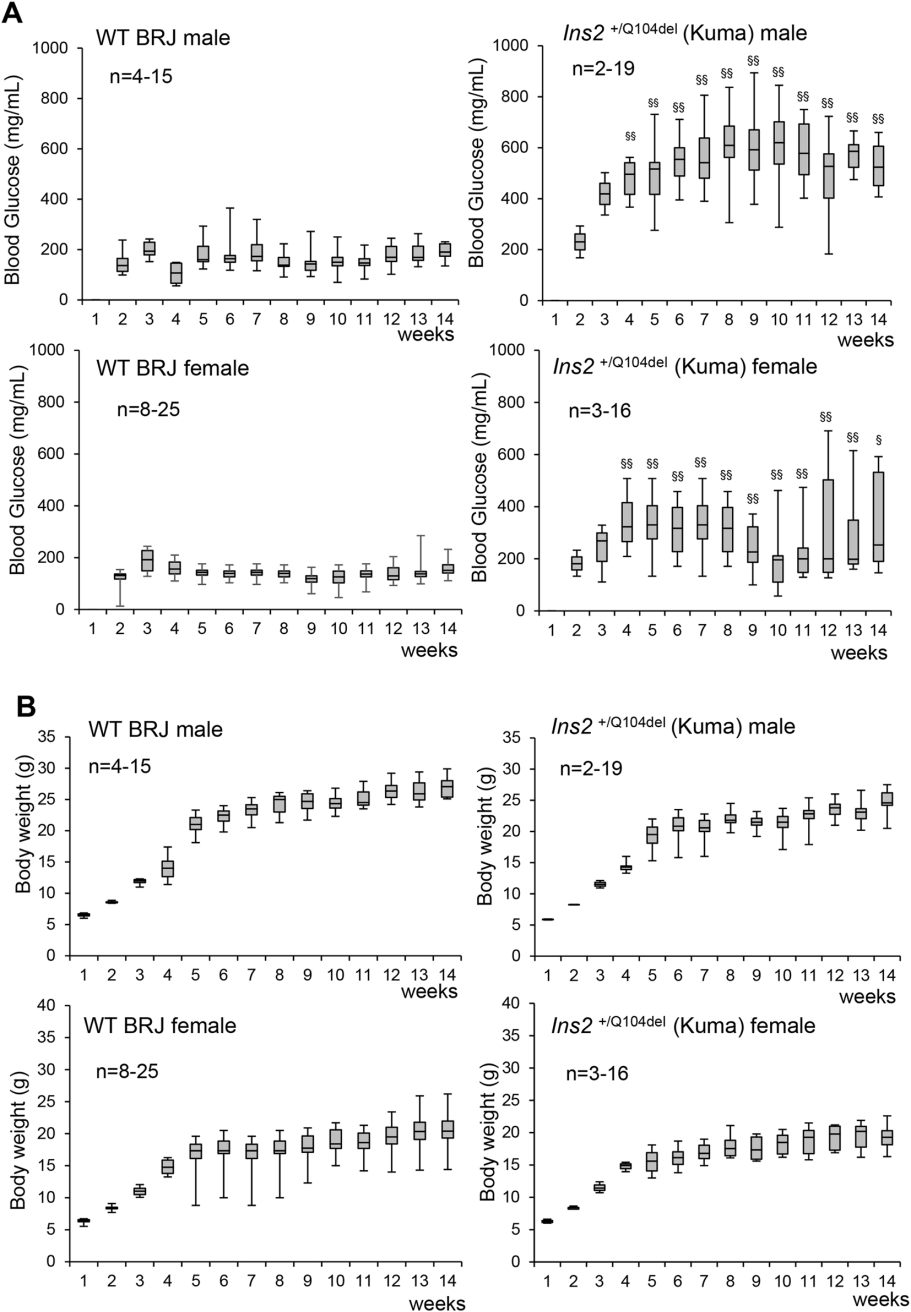
Kuma mice are non-obese diabetic. Age-dependent blood glucose and body weight of wild type (WT) BRJ and *Ins2*^+/Q104del^ (Kuma) mice are shown. (**A**) Blood glucose of the heterozygous Kuma (right panels) male (upper panels) or female mice (lower panels) showed significantly higher blood glucose compared to wild type BRJ mice (WT). (**B**) No differences in the body weights were observed between the WT BRJ and heterozygous Kuma mice. Data are shown as box and whisker plots. The upper quartiles, medians, lower quartiles are shown in the boxes. Upper and lower whiskers show maximum and minimum values. Significant differences in the values in Kuma heterozygous mice compared to those of the WT BRJ mice were analyzed by Kruskal–Wallis post-hoc Stell–Dwass’s test. Significant differences are shown as ^§^*P* < 0.05 or ^§§^*P* < 0.01.

The diabetes phenotype was examined with different ages in the wild type (WT) BRJ and Kuma mutant BRJ mice. Age-dependent non-fasting blood glucose was measured. Kuma BRJ mice revealed hyperglycemia in heterozygous males and females from young ages at 4 weeks and thereafter (Fig. 2A). Homozygous Kuma mutant male and female mice showed more severe phenotypes (KA, DS unpublished data). Female heterozygous Kuma mice showed milder hyperglycemia compared to that of the male Kuma mice. Age-dependent body weights of the male and female heterozygous Kuma mice revealed that Kuma mice are not significantly higher than those of the control wild type BRJ mice (Fig. 2B). We backcrossed Kuma mutant mice for more than 10 generations, and the phenotype is still observed, thereby excluding the possibility that the phenotype is due to off-target effects. The results indicate that Kuma mutant mice exhibit a non-obese permanent neonatal diabetes.

### Decreased insulin protein level and impaired glucose tolerance in Kuma mice

**Figure 3:**
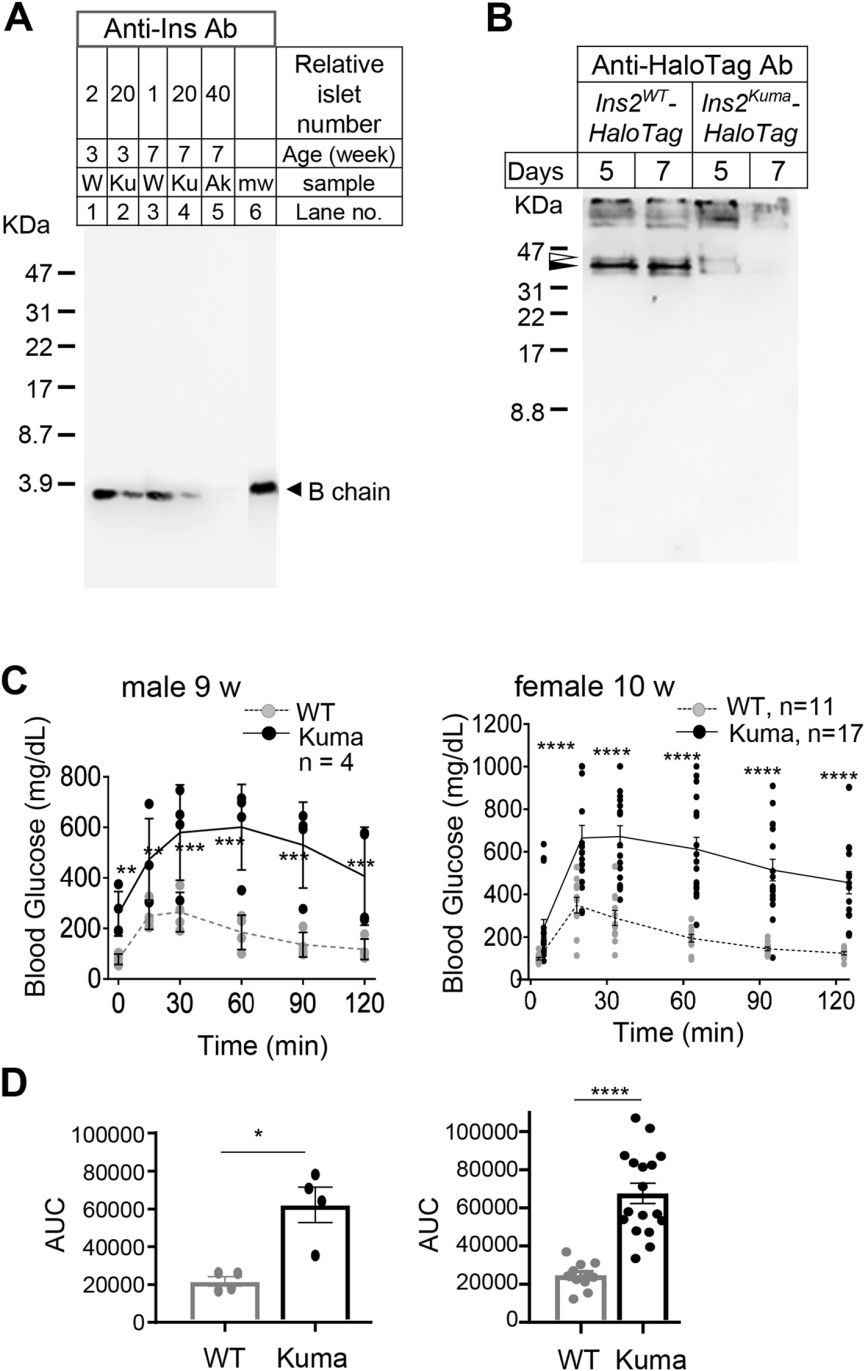
Decreased insulin protein levels and impaired glucose tolerance in Kuma BRJ mice. (**A**) Islets from WT, Kuma mutant mice, or Akita mice were isolated from 3- or 7-weeks-old, and whole-cell lysates were subjected to 16.5% SDS-PAGE (under reducing condition), and western blot analysis was performed using an anti-insulin B chain antibody. The ages of the mice used, and the relative islet numbers loaded are shown above each lane. *W* WT, *Ku* Kuma mutant, *Ak* Akita mutant islets, *mw*: molecular weight marker. (**B**) Whole-cell lysate of Min6 cells on day 5 and 7 post-transfection of adenoviral vectors encoding Ins2^WT^- or Ins2^Kuma^ -HaloTag fusion protein are subjected to non-reducing SDS-PAGE, and probed with an anti-Halo-Tag antibody. Open and closed triangles depict the two main bands that correlate to proinsulin and insulin, respectively. (**C**) Glucose tolerance tests are performed with WT BRJ and Kuma heterozygous males or females at 9- (N = 4) or 10-weeks-old (N = 17), respectively. Time-dependent blood glucose concentrations are shown. Kuma mouse: black circles with a solid line. WT BRJ mouse: gray circles with a broken line. Significant differences in the values in Kuma heterozygous mice compared to those of the BRJ mice were analyzed by two-way ANOVA followed by Sidak’s multiple comparisons test. (**D**) AUC (area under the curve) of the results shown in B are plotted, respectively. Impaired glucose tolerance in male and female Kuma heterozygous mice were observed. Individual data, together with mean ± SEM (standard error of the mean) are plotted. Significant differences were analyzed by Mann–Whitney’s U-test. *P < 0.05, **P < 0.01, ***P < 0.001, or ****P < 0.0001.

Since Kuma mice begin to reveal hyperglycemia from 4-week-old, we then isolated pancreatic islet whole cell lysates from 3- and 7-week-old WT and Kuma mice and performed western blot analysis to detect insulin. Kuma mutation does not harbor in the B chain. Therefore, we used an anti-insulin B chain antibody to identify insulin. As a result, 3-weeks-old Kuma mutant islets showed a significant reduction in insulin protein level, which further decreased at 7-weeks-old, compared to those from the WT islets. A low level of insulin was also observed in the Akita islets (Fig. 3A).

We then overexpressed a construct encoding Ins2^WT^- or Ins2^Kuma^ -HaloTag fusion protein in Min6 cells, a mouse beta-cell line. Min6 whole cell lysates were harvested on days 5 and 7 post-transfection and subjected to western blot analysis. Using an anti-HaloTag antibody, we could detect proinsulin (Fig. 3B, upper band depicted by an open triangle) and the processed insulin (Fig. 3B, lower band depicted by a closed triangle). WT proinsulin was rapidly processed to insulin. Kuma mutant proinsulin processing seemed to occurred, albeit the Kuma mutant forms of proinsulin- or insulin-HaloTag protein levels seem to be low on day 5 post-transfection, and more decreased on day 7. The results suggest that reduced stability or rapid degradation of Kuma mutant proinsulin and insulin is responsible for the reduction in insulin protein levels.

To examine the diabetic phenotype in detail, we performed glucose tolerance tests. The male (9-weeks-old) and female heterozygous Kuma (10-weeks-old) mice displayed impaired glucose tolerance compared to the WT BRJ mice (Fig. 3C). The areas under the curve (AUC) of the heterozygous Kuma mice in both males and females are significantly higher compared to the control WT BRJ mice (Fig. 3D), thereby indicating that these Kuma mice are glucose intolerant.

### Rescue of the hyperglycemia of the Kuma mutant by insulin administration

**Figure 4:**
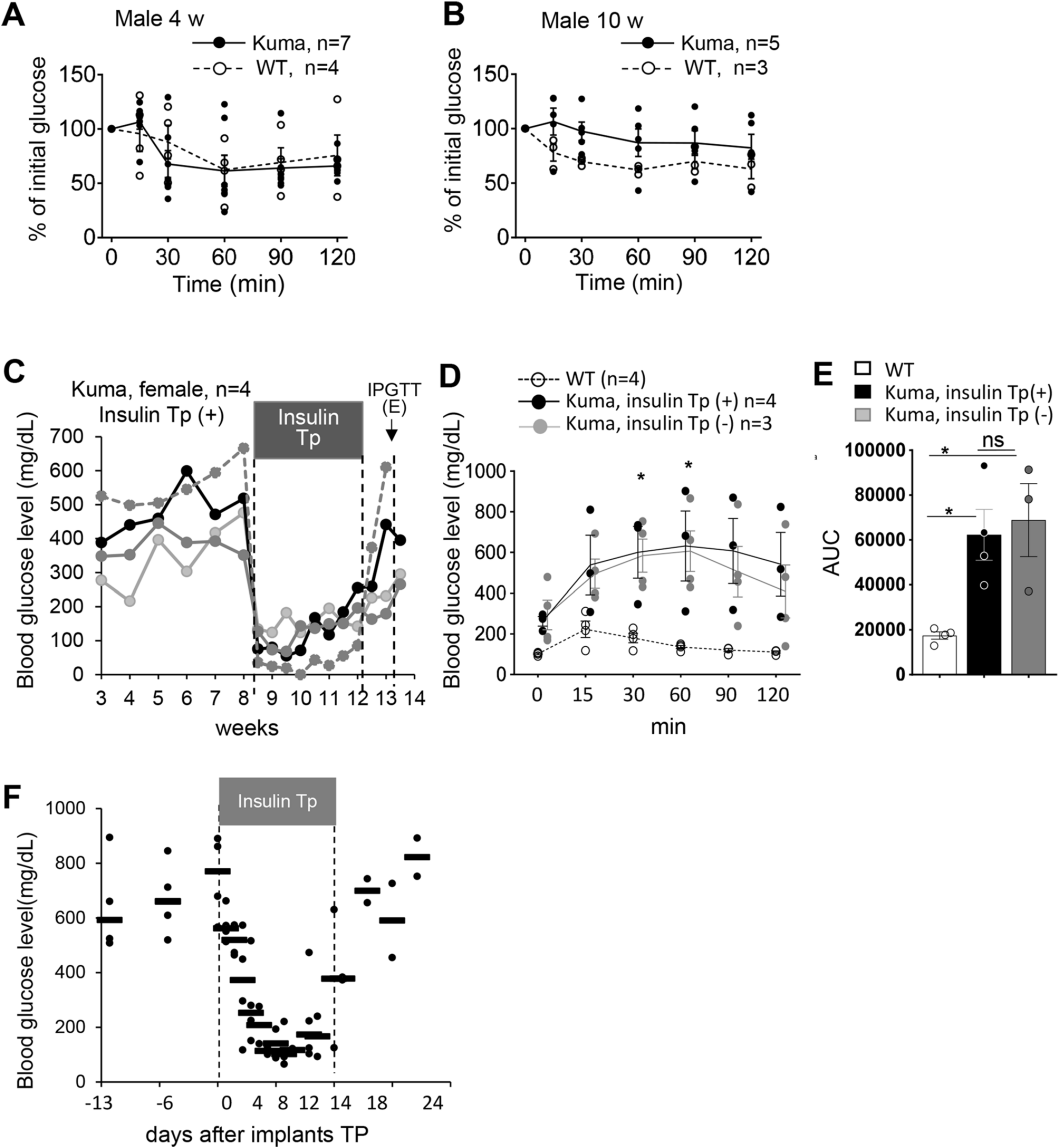
Normal insulin tolerance and reversal of blood glucose by insulin therapy in Kuma mutant mice. (**A**,**B**) Male WT BRJ and Kuma BRJ mice at 4-weeks-old (4w) (**A**) or 10-weeks-old (10w) (**B**) showed normal insulin tolerance. Plasma glucose levels were presented as the percent of change from glucose level at time 0. Kuma mouse: closed circles with solid lines; WT BRJ mouse: open circles with broken lines. (**C**–**E**) Kuma heterozygous mice treated with insulin implants are rescued from hyperglycemia. (**C**) Kuma heterozygous female mice have been measured for blood glucose showed hyperglycemia before treatment with insulin implants from 8.5-weeks-old. Upon insulin implant treatment, a rapid reversal of hyperglycemia was observed. Each line depicts an individual heterozygous Kuma mouse. Each plot indicates data from a single mouse. Gray box shows the time window that the mice are treated with insulin implants. After 1-month insulin implant treatment, the insulin implants were removed, a recurrence of hyperglycemia was observed. An arrow depicts the time point for IPGTT challenge. (**D**) Heterozygous Kuma mice received insulin therapy were challenged with IPGTT and revealed a recurrence of glucose intolerance (Insulin Tp (+), n = 4). IPGTT from Kuma heterozygous mice that did not receive insulin therapy (Insulin Tp (–), n = 3) were used as a control. (**E**) AUC of the results shown in (**D**). (**F**) Kuma heterozygous male mice at 10-weeks of age were measured for blood glucose from 2 weeks showed hyperglycemia, and were transplanted with insulin implants (Insulin TP: time window of Insulin implantation). Upon implantation, a rapid reversal of hyperglycemia was observed. After the removal of insulin implants, a recurrence of hyperglycemia was observed. Each dot represents an individual mouse. Each horizontal line depicts the median value of blood glucose. Scattered plots with individual results, together with means ± SEM, are presented. Significant differences were analyzed by Sidak’s multiple comparisons test (**A**,**B**) or Tukey’s multiple comparisons test (**D**,**E**). No differences were observed between the Kuma and WT mice (**A**,**B**), or between the Kuma ( +) and (−) insulin-treated animals (**D**). Significances between the WT and (+) or (−) insulin-treated animals are shown as *P < 0.05.

We then performed insulin tolerance tests. Since the phenotype in male Kuma mice is more severe than those of the female mice, we performed the experiments in male Kuma mice. The result revealed that in early life at 4-weeks-old or 10-weeks-old, male heterozygous Kuma mice showed normal insulin tolerance. However, the blood glucose levels showed more significant variations in the Kuma mutants than that of the WT BRJ control mice (Fig. 4A,B).

We then tried to administrate insulin to examine if blood glucose of these mutant mice could be normalized using insulin implants, which allowed a sustained release of insulin. After transplantation of insulin implants into female heterozygous Kuma mice at 8.5-weeks of age, hyperglycemia of the Kuma mice was rapidly normalized, sustained for 1 month. Upon removal of insulin implants after 1 month, a recurrence of hyperglycemia was observed (Fig. 4C), and Kuma mutant mice still showed impaired glucose tolerance, which was not significantly different from those of the untreated group (Fig. 4D,E). The results show that hyperglycemia and impaired glucose tolerance are reversible and could be normalized by insulin administration. Another experiment transplanting insulin implants into male heterozygous Kuma mice at 10-weeks of age also rescued the hyperglycemia (Fig. 4F).

### Reduced islet areas in Kuma mice

**Figure 5:**
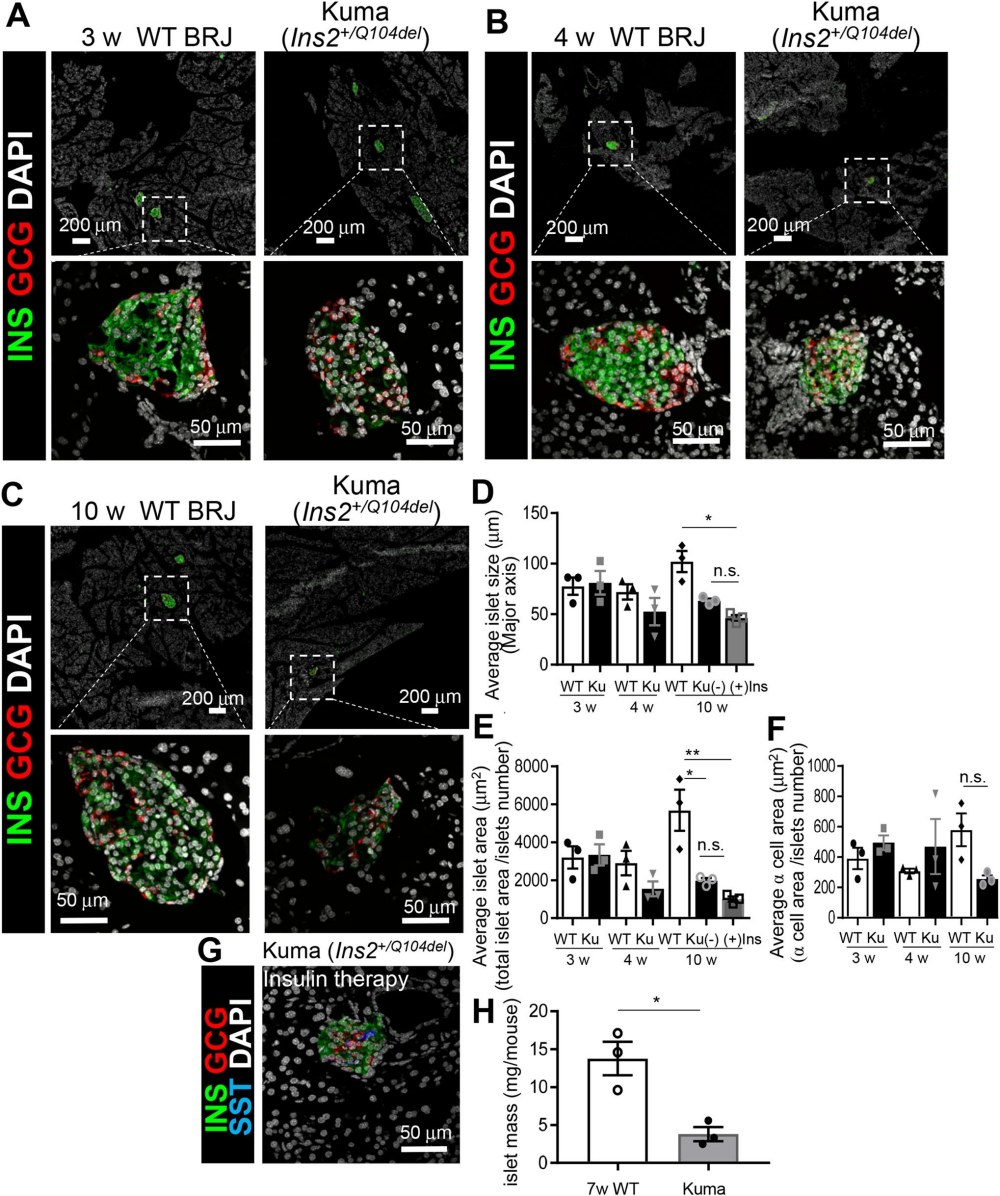
Decreased islet area and islet mass in Kuma mice. Pancreas dissected from male WT and Kuma mice at 3w (**A**), 4w (**B**) or 10w (**C**) of age are subjected to immunohistochemistry to detect the insulin (INS, green) and glucagon (GCG, red) expression in the islets. Islet size (**D**), beta-cell area (**E**), and alpha-cell area (**F**) are quantified and compared between the WT and Kuma heterozygous mice at 3w, 4w, and 10w, with or without treatment with insulin implants from 4 weeks. DAPI, 4′,6-diamidino-2-phenylindole, counterstaining of the nuclei (white). (**A**) WT BRJ mice exhibited insulin-expressing islets, with glucagon-expressing alpha-cells, exist in the periphery of the islets. In the Kuma mutant mice at 3 w, glucagon-expressing alpha cells seem not restricted to the periphery of the islets. (**B**) In Kuma mice at 4 w, the size of islets and islet mass tend to be smaller, and insulin-expressing cells became fewer. Glucagon-expressing alpha-cells are scattered inside the islets. Representative pictures are shown. (**C**) In Kuma mice at 10 w, islet sizes and beta-cell mass are significantly reduced compared to that of the WT. (**D**–**F**) Quantitative measurements of islet size (long axis length, μm) (**D**), islet area (μm^2^) (**E**), or alpha-cell area (μm^2^) (**F**), in WT or Kuma mutant mice at 3, 4, and 10 w. Kuma mutant without or with insulin implant treatment, Ku (+), (−) Ins. (**G**) Kuma mutant at 10 w treated with insulin implant for 4 weeks still showed a small islet area. (**H**) Islet mass from WT and Kuma mutant at 7 w. (**A**–**C**) Upper panels, low magnifications. Scale bars represent 200 μm. Lower panels, high magnifications. Scale bars represent 50 μm. Scattered plots with individual results together with mean ± SEM are presented. Significant differences were analyzed by two-way ANOVA, followed by Tukey’s multiple comparisons test (**D**–**F**), or unpaired two-tailed Student’s t-test (**H**), and shown as *P < 0.05, **P < 0.01. N = 3.

We then examined the islet architectures of the Kuma mice. WT BRJ, heterozygous Kuma mice at 3-, 4- and 10-weeks-old were sacrificed, and the pancreas was excised and analyzed. Immunohistochemical analyses were performed with antibodies against insulin and glucagon. Representative pictures of islets in Kuma mice and WT mice are shown in Fig. 5. At 3-weeks of age, we observed no significant differences in islet morphologies between the WT and heterozygous Kuma mutant mice. However, the Kuma mutant mice seemed to show a decreased insulin expression levels (Fig. 5A). At 4-weeks of age, islet size and insulin-positive beta-cell area in heterozygous Kuma mutant tended to be smaller but did not statistically differ from those of the WT (Fig. 5B,D,E). At 10-weeks of age, the average islet size and islet area became significantly smaller in the heterozygous Kuma mutant compared to those in the WT mice (Fig. 5C–E). On the other hand, the alpha-cell area was not significantly different between the WT and heterozygous Kuma mutant mice (Fig. 5F).

Since Kuma mice treated with insulin implants showed a reversal of hyperglycemia (Fig. 4C–E), we then examined if the endogenous islets were affected using the pancreas dissected from Kuma mice treated with insulin implants for 4 weeks from 6-weeks of age. The results revealed that islet sizes and islet areas were not significantly different between those of the insulin implant treated and untreated Kuma mice at 10-weeks of age (Fig. 5D,E,G). The results suggest a decrease in islet mass occurs in the Kuma mutant. We then isolated islets from 7-weeks old WT and Kuma mice and measured islet mass. We found that the islet mass was significantly decreased in 7-weeks-old Kuma mutant compared to that of the WT mice.

### Marked reduction in the number and size of the matured insulin granules in the Kuma mutant mice

**Figure 6:**
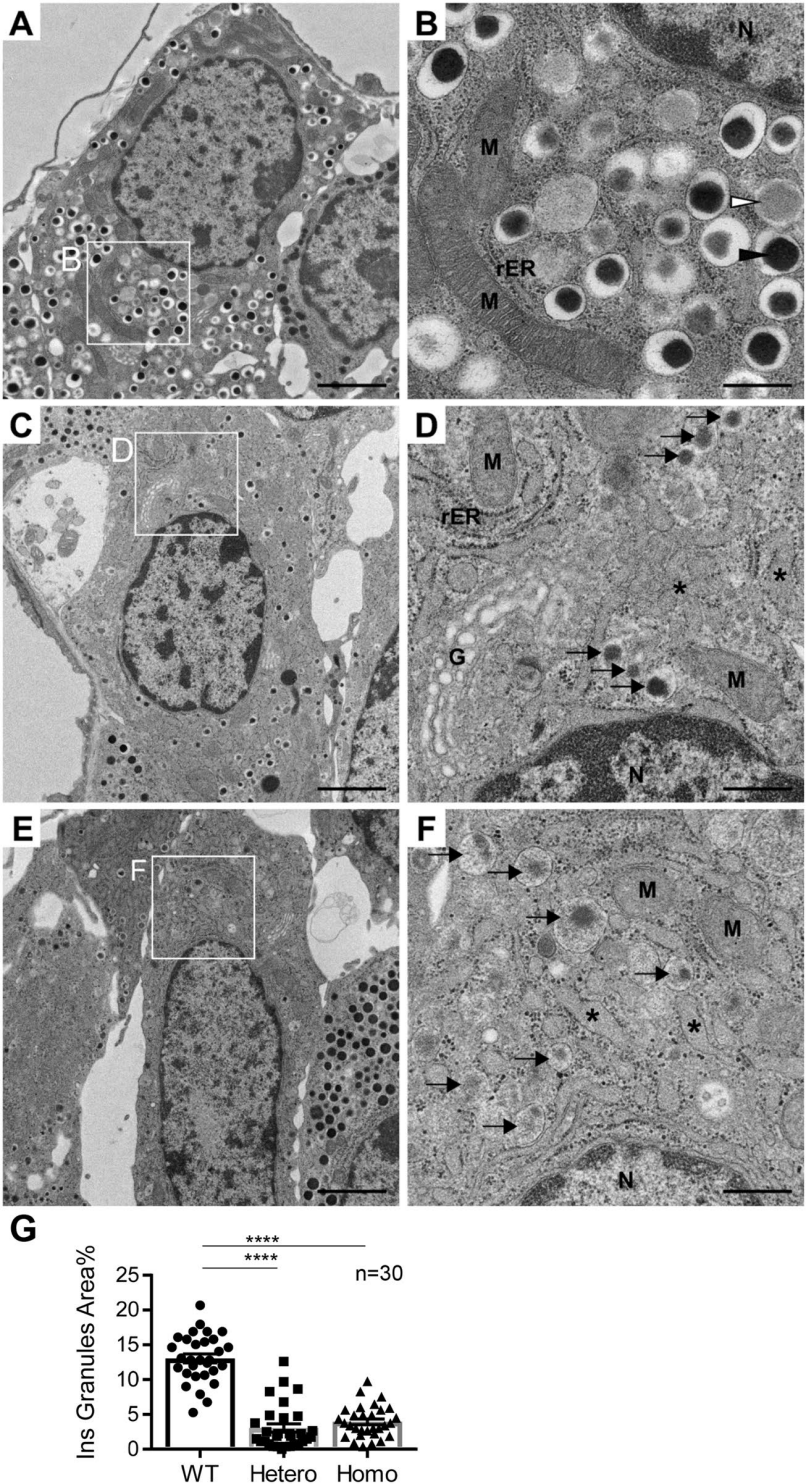
Ultrastructural analysis of beta-cells revealed a marked reduction in beta-cell granule number and size in Kuma mice. Electron microscopy of beta-cells in 5 w WT (**A**,**B**), heterozygous (**C**,**D**), and homozygous (**E**,**F**) Kuma mutant mice. (**B**,**D**,**F**) represent higher magnification of the boxed regions in (**A**,**C**,**E**), respectively. Bars are 2 μm (**A**,**C**,**E**) or 500 nm (**B**,**D**,**F**). (**G**) Quantification of insulin granules area in WT, hetero, and homo Kuma mice. One-way ANOVA, Dunn’s multiple comparisons test. N = 30, ****p < 0.0001. White and black arrowheads represent immature and mature insulin granules in a beta-cell of WT, respectively. Note that beta-cells from Kuma mutant mice show immature insulin granules with a lower number and smaller size (arrow), dilated ER structures (asterisks). *G* golgi, *M* mitochondria, *N* nucleus, *rER* rough-surfaced endoplasmic reticulum.

We then performed electron microscopy to examine the details in the ultrastructure of the beta-cells in Kuma mice. In 5-weeks-old WT BRJ mice islets, many dense core mature insulin granules in the beta-cells were observed (Fig. 6A,B). By contrast, Kuma heterozygous (Fig. 6C,D) or homozygous beta-cells (Fig. 6E,F) revealed a marked reduction in numbers and sizes of the insulin granules. The cores of the insulin granules in the Kuma mutants are less electron-dense compared to those of the WT mice, demonstrating characteristics of immature beta-cells (Fig. 6C–F). In the Kuma mutant mice, endoplasmic reticulum (ER) show dilated vesicular morphologies (Fig. 6D,F, asterisks). Quantification studies revealed a significant reduction in insulin granule area in proportion to the beta-cells area in the Kuma mutants compared to that of the WT mice (Fig. 6G). Our results revealed that the beta-cells in Kuma mutants scarcely possess mature insulin granules and exhibiting devastating changes in ER architectures.

### No increase in gene expression levels of the ER stress marker genes in the Kuma mice

**Figure 7:**
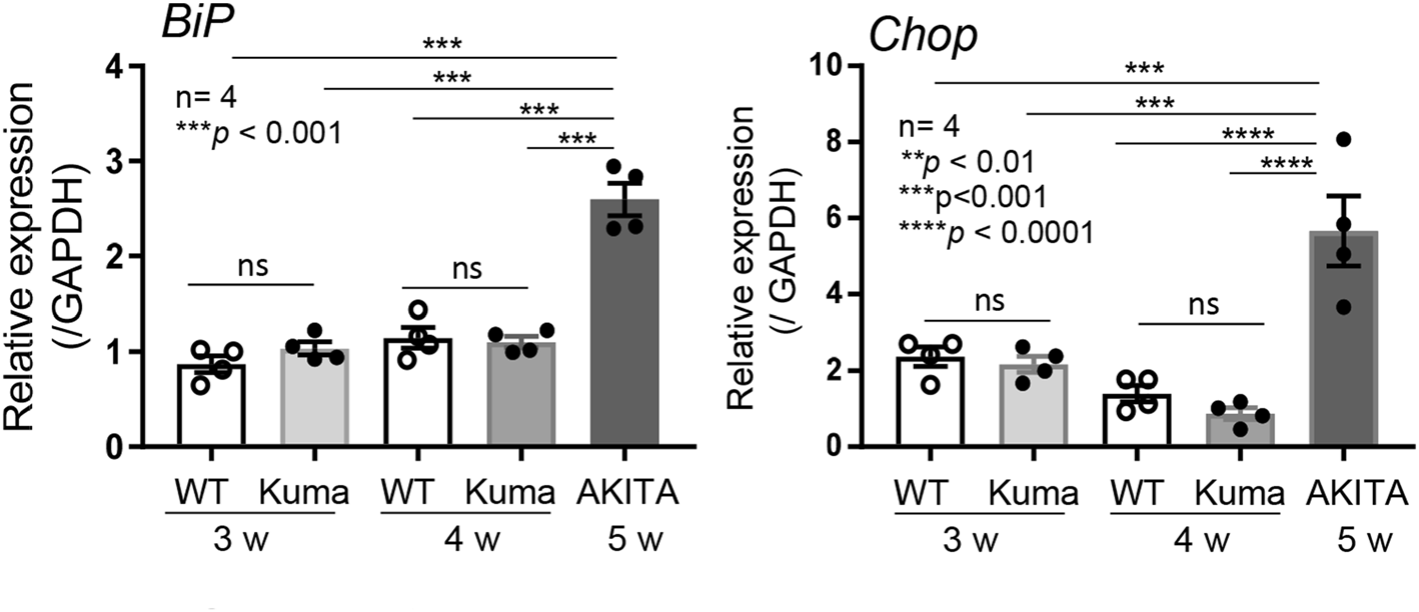
No increase in the expression levels of ER stress marker gene in the Kuma mice. *BiP* and *Chop* expression levels in WT, Kuma, and Akita mice were examined by real-time PCR. *BiP* (left) and *Chop* (right) expression levels increased in 5 w Akita islets, but not in 3 w or 4 w WT or Kuma islets. Significant differences were analyzed by one-way ANOVA, Tukey’s multiple comparisons test. N = 4, ***P < 0.001, ****P < 0.0001.

In the Akita mice, it is reported that the expression levels of an ER chaperone, *Binding of immunoglobulin protein* (BiP), and an ER stress-associated apoptosis factor *CCAAT/enhancer-binding protein homologous protein* (*Chop*) are upregulated^13^. The targeted disruption of *Chop* delayed the onset of diabetes in Akita mice^13^. To gain insight into the decreased beta-cell mass, we then analyzed the expression levels of *BiP* and *Chop* in the Kuma mutant mice. Islets from WT and Kuma mice at 3- and 4-weeks-old and from Akita mice at 5-weeks-old were used for analysis. Both *BiP* and *Chop* expression levels were significantly upregulated in the islets from Akita mice compared to that in the WT. In contrast, the expression levels of *BiP* and *Chop* were not upregulated in the Kuma mutant islets (Fig. 7). The results suggest that ER stress is not increased in the Kuma mice, and might not be the cause of the reduction in beta-cell mass.

## Discussion

Neonatal diabetes is early-onset diabetes that is diagnosed within the first 6 months of life in humans and could be subdivided into the persistent, permanent neonatal diabetes and the relapsing–remitting transient neonatal diabetes^1, 2, 14^. Monogenic mutations, such as activation mutations in *KCNJ11* or *ABCC8*, the genes encoding the potassium ATP-sensitive (K_ATP_) channel subunits Kir6.2 or SUR1, respectively, or heterozygous mutations in the *preproinsulin* (*INS*) gene, are known to be the main causes that associate with permanent neonatal diabetes^1, 2^. *INS* gene mutation resulted in insufficient insulin secretion that leads to hyperglycemia. More than 51 insulin-gene mutations have been identified to cause monogenic diabetes in human^15^. Most of the mutations were predicted to disrupt the folding of the proinsulin molecule, which results in ER stress and beta-cell apoptosis^3, 15^.

In this study, we generated an Ins2 p.Q104del (Kuma) mutation in a severe immune-deficient BRJ mouse model^8^. Kuma mutant mice were non-obese, and showed normoglycemia at the time of birth but developed diabetes at a young age of 4-weeks-old. This mutation is autosomal dominant inheritance and resembles the diabetes of permanent neonatal diabetes in humans. The Kuma heterozygous mice showed a reduction in insulin protein levels from early life at 3-weeks-old. Morphological examination of the pancreas revealed that the beta-cell area was initially unaltered at birth but seemed to become reduced gradually from 4-week-old, and later at 10-weeks of age, a significantly reduced averaged islet size, islet area, and islet mass were observed. In contrast, the alpha-cell area did not show marked reduction. Ultrastructural morphology observation of beta-cells in 5-weeks-old Kuma mutant mice revealed a marked reduction in the area of the insulin granules. In the Kuma mice, insulin resistance was not evident at 10-week-old. We treated Kuma mice with insulin implants for 4 weeks and found that this could rescue the mice from hyperglycemia. Therefore, Kuma mutant mice would be useful as a model for studies of interspecies transplantation for the cure of insulin-dependent diabetes. The Q104 in *Ins2* gene is well conserved across species. Our report is by far the first that report this mutation to trigger early-onset diabetes.

A transgenic pig model, generated by introducing an *INS*^C94Y^ transgene, also showed beta-cell dysfunction in a dominant-negative manner^7^. In Akita mice, it is reported that the accumulation of the misfolded proinsulin might cause a prolonged activation of the unfolded protein response signaling pathways, lead to chronic ER stress and cause the organelle dysfunction, thereby inducing apoptosis of beta-cells^16, 17^. We did not observe an increase in the expression levels of the ER stress marker genes, *BiP*, or *Chop*.

We observed low Ins2^Kuma^ protein levels in the beta-cells of the Kuma mutants. We then overexpressed WT and Kuma Ins2-HaloTag fusion proteins in Min6 cells and revealed reduced protein levels of the Kuma mutant forms compared to those of the WT. Our results suggest low stability of the Kuma proinsulin and insulin mutant proteins.

To investigate the underlying mechanism of the reduced beta-cell mass in Kuma mutant, we overexpressed Ins2^WT^ or Ins2^Kuma^ tagged with 3xFlag-T2A-mCherry. We observed a significant increase in the proportion of cleaved caspase3 among the *Ins2*^*Kuma*^-expressing beta-cells in a disperse primary islet culture (Fig. S1B). The results suggest that an increase in apoptosis might be one of the mechanisms that lead to beta-cell dysfunction and decreased beta-cell mass in Kuma mutant mice.

It was reported that in the Akita mice, inhibition of mTORC1 that governs postnatal beta-cell growth and differentiation leads to impaired expansion of beta-cell mass^18^. Another report using induced pluripotent stem cell (iPSC) derived from patients with neonatal diabetes carrying mutations in the insulin gene revealed that the derived insulin mutant beta-like cells exhibited reduced proliferation^19^. A reduced expansion of the mutant beta-cells and increased apoptosis might occur, which leads to the reduced beta-cell mass in the Kuma mutants.

Akita mice develop impaired glucose tolerance and are shown to exhibit insulin resistance at older ages, in which hyperglycemia is suggested to play a role^20^. A previous report shows that early insulin therapy prevents beta-cell loss in a Munich *Ins2*^C95S^ permanent neonatal diabetes mouse model^21^. However, our insulin therapy results suggest that insulin therapy from 10-weeks of age exerted no significant effects on the endogenous beta-cell mass itself.

We believe that the Kuma mutant will serve as an attractive model not only for the studies of the *Insulin* gene and the molecular mechanism for the maintenance of beta-cell mass, and the development of insulin resistance under hyperglycemia. Induction of insulin-deficient diabetes, such as pancreatectomy, is highly invasive. Chemical diabetes induction, such as streptozotocin treatment, displayed variable outcomes depending on mouse strains and ages and is reversible at low doses^22–24^. In contrast, the Kuma mice displayed stable diabetes, similar to Akita mice^25^. Kuma mutation in BRJ mice is useful as a good model for testing the functionality of transplanted beta-cells generated from human iPS cells. The Kuma mutation is well conserved across different species and could be used for generating permanent early-onset diabetes model in other experimental animals.

## Material and methods

### The institutional committee approving the experiments

All experiments were approved by the Institutional Committee for Animal Research in Tokyo Institute of Technology and Institutional Committee for Animal Research in Kumamoto University.

### Animals

BALB/c strain of Rag-2/Jak3 double-deficient (BRJ) mice^8^ were used. WT BRJ or Kuma (*Ins2* ^+/Q104del^) BRJ mice were housed and monitored in our animal research facility according to institutional guidelines in Kumamoto University or Tokyo Institute of Technology. All methods were performed following the institutional guidelines in Kumamoto University or Tokyo Institute of Technology. Kuma mouse is maintained either by crossing heterozygous females with wild type male BRJ mice (only the early generations) or by in vitro fertilization.

Mice were allowed free access to food and water except when being fasted. Genotyping was performed using the genomic polymerase chain reaction (PCR) analysis of the *Insulin* (*Ins*) *2* gene using the primer pair indicated in Fig. 1C. The amplified PCR fragments were subjected to Pvu II restriction enzyme cut. Mutant mice were identified to be resistant for Pvu II. Fasting blood glucose levels and body weights were measured from 2 weeks of age onwards in mice that had been fasted for 2 h using a portable glucose monitor (Life check sensor, Gunze, Tokyo, Japan) or blood glucose meter ANTSENSE III (Horiba, Kyoto Japan), by making an incision in the tail.

### Genomic PCR for genotyping of Kuma mutation

Genotyping was performed by PCR amplification, with DNA extracted from mouse tails. For genotyping, PCR analysis of the *Ins2* gene was performed using the following primer pair: 5′-AGGGGTCCTTGGTGGTAGTAA CTT-3′ and 5′-CAGGAGAAAACTGGGGATGC-3′. The amplified fragment was cut with Pvu II (the recognition site 5′-CAGCTG-3′) and subjected to analysis using 1% agarose. WT allele gives bands at 248 bp and 425 bp, whereas the mutant allele gives a band at 670 bp (Fig. 1B).

### Western blot analysis for detection of Insulin proteins

Mouse islets from 3–4-week-old WT BRJ or Kuma BRJ mice were isolated as described previously^25^. Isolated islets were handpicked and lysed with sample buffer (75 mM Tris–HCl at pH 6.8, 1% SDS, 10% glycerol, 2.5% sucrose containing 1 µg/mL BSA), added with 5% 2-mercaptoethanol, boiled at 95 °C for 5 min. Separation of proteins was performed using a Tris-Tricine SDS-PAGE (Tricine-sodium dodecyl sulfate–polyacrylamide gel electrophoresis) system as previously described with modifications^26^. Briefly, lysates were run through a 16.5% Mini-PROTEAN Peptide Gel (Bio Rad, California, USA), blotted to a PVDF membrane (Bio-Rad, California, USA), and probed overnight at 4 °C with anti-Insulin antibodies (Rabbit monoclonal Ab clone c27c9, Cell Signaling Technology). The secondary antibody used was Peroxidase conjugated AffiniPure Goat Anti-Rabbit IgG (H + L) (Jackson). Western blots were developed with enhanced chemiluminescence and detected using FUSION-SOLO.4S.WL (M&S instruments, Tokyo, Japan).

### Western blot analysis for detection of Ins2-HaloTag fusion proteins

Virus-infected Min6 cells were lysed with sample buffer as described above, and separated using a Tris-Tricine SDS–PAGE as described^27^. The lysed islets samples were resolved using a 16% polyacrylamide gel, blotted to a PVDF membrane (Millipore, Germany), and probed overnight at 4 °C with antibodies against Halo-Tag (Promega). The secondary antibody used was Peroxidase AffiniPure Goat Anti-Mouse IgG (H + L) (Jackson). Detections were performed using FUSION-SOLO.4S.WL (M&S instruments, Tokyo, Japan).

### Adenoviral vector construction for Ins2-HaloTag fusion protein expression

The cDNA was generated from reverse transcription using RNA from WT BRJ or Kuma BRJ mouse islets. The cDNA encoding *Ins2*^*WT*^ or *Ins2*^*Kum*a^ were amplified from WT BRJ or Kuma BRJ mouse islets cDNA, using specific primer sets as follows. *Ins2*^*WT*^: Ins2 Fw 5′-ATCCGGTACCGAATTCATG GCCCTGTGGATGCGC-3′ and Ins2 Rev 5′-GTCTTTGTAGTCCATGTTGCAGTAGTTCTCC AGC-3′ primers. *Ins2*^*Kum*a^: Ins2 Fw and Kuma Rev 5′-GTCTTTGTAGTCCATGTTGCAGTAG TTCTCCAGGTAGAGG G-3′primers. The *Ins2*^*WT*^ or *Ins2*^*Kum*a^ cDNAs (excluding the stop codon) were fused with HaloTag or 3xFlag-T2A-mCherry cDNA. These gene cassettes were inserted into the multiple cloning sites of the pENTR1A vector (Invitrogen), respectively. The plasmids thus obtained were named as pENTR1A-Ins2-Halo (*Ins2*^*WT*^ or *Ins2*^*Kum*a^: Genbank MT671463 or MT671464) and pENTR1A-Ins2-3Flag (*Ins2*^*WT*^ or Ins*2*^*Kum*a^: Genbank MT671461 or MT671462). LR recombination reactions were performed to transfer the gene cassette to the pAd⁄CMV⁄V5**-**DEST vector using the Gateway LR Clonase II Enzyme Mix (Invitrogen), thus obtained the pAd⁄CMV⁄V5**-**Ins2-HaloTag or pAd⁄CMV⁄V5-Ins2-3xFlag-T2A-mCherry plasmids. All the plasmids were amplified in XL10 *E. coli* (Invitrogen) and linearized using Pac I (Toyobo) before use.

### Generation of adenovirus

The 293A cells were cultured in Dulbecco’s modified Eagle’s medium (DMEM) containing glucose (4.5 g/L), penicillin (200 U/mL), streptomycin (100 µg/mL), and 10% fetal bovine serum (FBS) at 37 °C, 5% CO_2_ incubator. After seeded for 24 h, the cells were transfected with pAd⁄CMV⁄V5**-**Ins2-HaloTag or pAd⁄CMV⁄V5**-**Ins2-3xFlag-T2A-mCherry plasmids, using Lipofectamine 3000 (Invitrogen) according to the manufacturer instructions. Adenoviruses from the culture supernatants of HEK 293A cells were collected and stored at -80℃ until use. Virus titers were determined by plaque assay using serial dilution.

### Intraperitoneal glucose tolerance test (IPGTT)

IPGTT was performed 9 weeks after transplantation. Mice fasted for 16 h were used. Blood glucose levels were measured before (0 min) and at 15, 30, 60, 90, and 120 min after intraperitoneal administration of 25% glucose solution (Wako, Osaka, Japan) at 2 g/kg bodyweight.

### Intraperitoneal insulin tolerance test (ITT)

We performed an insulin tolerance test as an assessment of insulin sensitivity. Mice were fasted for 6 h and then administered with an intraperitoneal injection of insulin solution (2.0 U insulin/kg body weight, HUMULIN R (regular human insulin injection, USP [rDNA origin]), Eli Lilly, Indianapolis, IN). Glucose levels were monitored as described above in the IPGTT.

### Insulin therapy

Insulin treatment was performed via implantation of Linbit sustained-release insulin implants (approximately 0.1 U/d/implant; Linshin Canada, Inc., Scarborough, Canada)^22^. These insulin implants are made of an admixture of insulin and micro-recrystallized palmitic acid, allowing a sustained release of insulin that achieves a more constant blood glucose level than daily insulin injections. After brief isoflurane (Merck animal health, New Jersey, USA) anesthesia, insulin implants were introduced by subcutaneous implantation in the nape of the neck, at a rate of 2 implants for < 20 g or 3 implants for 20–25 g bodyweight. Serum glucose levels were monitored, and insulin-treated animals were used in experiments after 2 weeks of insulin therapy, as indicated. Four weeks after insulin therapy, insulin implants were removed.

### Immunohistochemistry

Tissue samples were fixed with 4% formaldehyde, cryoprotected with 30% sucrose, and cut into 10 μm thick sections. The following antibodies were used: guinea pig anti-insulin (Dako Denmark A/S, Glostrup, Denmark; 1:1,000), mouse anti-glucagon (Sigma-Aldrich; 1:1,000). Alexa Fluor 488 donkey anti-guinea pig IgG (Life Technologies; 1:1,000), and Alexa Fluor 568 donkey anti-mouse IgG (Biotium; 1:1,000). Tissue sections were counterstained with 4,6-diamidino-2-phenylindole (DAPI; Roche Diagnostics, Basel, Switzerland).

### Electron microscopy

Electron microscopic analysis was performed as previously described^28^. Briefly, anesthetized mice were fixed by cardiac perfusion with a fixative containing 2% (w/v) paraformaldehyde and 2% (w/v) glutaraldehyde in 0.1 M phosphate buffer (PB) (pH 7.2). Samples were processed for post-fixation with 1% OsO4, dehydration with a graded series of ethanol and embedding in Epon812 (Oken Shoji). Ultrathin sections were cut with an ultramicrotome UC6 (Leica Microsystems), stained with uranyl acetate and lead citrate, and examined with a Hitachi HT7700 electron microscope (Hitachi, Tokyo, Japan). After printing the original magnification on projection papers, we estimated the % area of the insulin granule in beta-cells by point counting, using a double-lattice test system of 1.0-cm spacing^29^.

### Real-time PCR analysis

RNA was extracted from cells using RNeasy mini-kit (Qiagen, Hilden, Germany) and then treated with DNase I (Qiagen, Hilden, Germany). Complementary DNA was synthesized from 1 μg of total RNA using PrimeScript RT Reagent Kit (Takara Bio, Shiga, Japan). For real-time PCR analysis, the mRNA expression was quantified with SyberGreen on an ABI 7500 thermal cycler (Applied Biosystems, Foster City, CA). The level of each gene expression was normalized with that of β-actin. The PCR conditions were as follows: denaturation at 95 °C for 15 s, annealing and extension at 60 °C for 60 s, for up to 40 cycles. Each measurement was normalized to β-actin for each sample by subtracting the average β-actin. The relative expression levels were determined using the standard curve method. Primer design is as follows. *Bip* Fw 5′-AGGACAAGAAGGAGGATGTGGG-3′, Rev 5′-ACCGAAG GGTCATTCCAAGTG-3′; *Chop* Fw 5′-TTCACTACTCTTGACCCTGCGTC-3′, Rev 5′-CAC TGACCACTCTGTTTCCG TTTC-3’.

### Dispersed islet cell culture and adenovirus vector-mediated gene transduction

Dispersed islet cells were resuspended in RPMI-1640 containing penicillin (200 U/mL), streptomycin (100 µg/mL) and 10% FBS, and were plated onto culture dishes, at a density of 3 × 10^4^ cells/cm^2^. One day after plating, the virus-containing medium was added. On the following day, islet cells were extensively washed with RPMI-1640 with 10% FBS. The virus-treated islet cells were subjected to immunostaining, fluorescence observation, or western blot analysis.

### Statistics

Data are presented as mean ± SEM (standard error of the mean), medians in box and whisker plots, or plotted with individual data. The normal distribution of the data was confirmed using the Kolmogorov–Smirnov test. In Fig. 2, as no normal distribution could be demonstrated, we applied the nonparametric Kruskal–Wallis with the post-hoc Stell-Dwass’s test. Significant differences are shown as ^§^*P* < 0.05, ^§§^*P* < 0.01. Figures 3C, 4A,B, 6 Sidak’s multiple comparison test. Figure 3D, Mann–Whitney U-test. Figures 4D,E, 5D–F and 7 Tukey’s multiple comparison test. Figures 5H, S1B, unpaired two-tailed Student’s t-test was applied. Significances are shown as *P < 0.05, **P < 0.01, ***P < 0.001, ****P < 0.0001, and are indicated in the figure legends. GraphPad Prism (GraphPad Software, 7.05) was employed for the statistical analysis.

## Supplementary information

Supplementary information

## Data Availability

All data generated or analyzed during this study are included in this published article (and its Supplementary Information files). The plasmid sequence data generated during this study are available at GenBank database under the following accession numbers: MT671461, MT671462, MT671463 and MT671464.
