# Supplementary material for: Insulin2Q104del (Kuma) mutant mice develop diabetes with dominant inheritance
Source: Sci Rep. 2020 Jul 22;10:12187. doi: 10.1038/s41598-020-68987-z (PMC7376009; doi:10.1038/s41598-020-68987-z)
Supplement: Supplementary file 1 — Supplementary information [file 41598_2020_68987_MOESM1_ESM.pdf]

Supplementary information

“*Insulin2* ^{Q104del} (Kuma) Mutant Mice Develop Diabetes with Dominant Inheritance.”

Daisuke Sakano, Airi Inoue, Takayuki Enomoto, Mai Imasaka, Seiji Okada, Mutsumi Yokota, Masato Koike, Kimi Araki, Shoen Kume

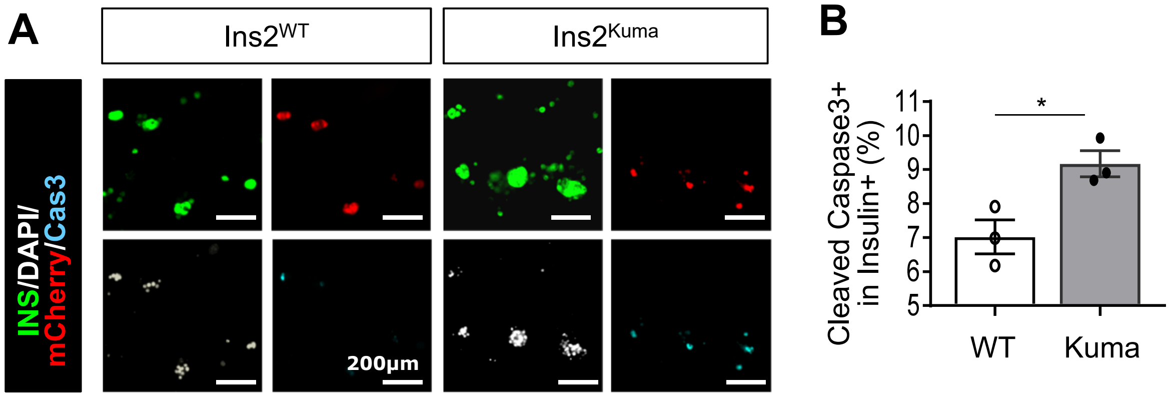
A
Ins2WT
Ins2Kuma
INS/DAPI/mCherry/Cas3
200μm
B
Cleaved Caspase3+ in Insulin+ (%)
*
11
10
9
8
7
6
5
WT
Kuma

Figure S1 *Ins2*^{Kuma} overexpression in islet cells increases beta-cell apoptosis

Overexpression of genes encoding *Ins2*^{WT} (B, left panels) or *Ins2*^{Kuma} -3xFlag-T2A-mCherry fusion proteins (B, right panels). Insulin-expressing beta-cells: green. Activated caspase 3, blue. mCherry, red. mCherry-positive cells demonstrate cells overexpressed *Ins2*^{WT} or *Ins2*^{Kuma}. Cleaved caspase 3+ cells among insulin+ cells (%) in *Ins2*^{WT} or *Ins2*^{Kuma} overexpressed Min6 cells. Significant differences were analyzed by Student's *t*-test. $^{*}P < 0.05$, N = 3.
